# Maurits Sterkenburg, an intriguing artist

**DOI:** 10.1017/S2045796021000780

**Published:** 2022-01-18

**Authors:** Elvira van Eijl

**Affiliations:** ArtEZ Institute of the Arts, Amsterdam, the Netherlands

Twice a year I am asked to join the election committee of Special Arts in Amersfoort in the Netherlands to choose new candidates for their collection and their art borrowing service. Special Arts (www.specialarts.nl) is a nonprofit umbrella organisation that gives ‘outsider artists’ and artists who are not ‘officially accepted as artists’ the possibility to show their work to the public, get advice and help with their exhibitions.

Artists from all over the country take part; they introduce their work and talk about their concepts. As a curator of the Art Brut Biënnale (www.artbrutbiennale.nl) I am always looking forward to these meetings because to me it is pure joy to meet interesting new artists who because of their ‘otherness’, are unfortunately forced to work in the ‘shadows’ of the official artworld. Our Art Brut Biënnale is a nonprofit organisation and a large international platform for Art Brut artists to exhibit their work, get in touch with each other and get acquainted with an audience who are interested in their work.

## The Kolozaieken

Five years ago a broad and somewhat shy young man with a bucket hat on his half long hair entered the reception room and started to unpack his artworks. We all felt the temperature and the tension in the room rising. My colleagues were as enthusiastic as I was. We had never seen such peculiar sculptures before. In front of us were a dozen robotlike figures displayed, the size of dolls and made out of colourful wool. They were called ‘Kolozaieken’, a combination of the words colossal and colourful, like a mosaic. Recently, he also invented an international name for them ‘Androikolo’, because that is easier to pronounce outside the Netherlands.

The Kolozaieken appear in different shapes, big and small, vulnerable and strong, with dreadlocks and sometimes with many arms and legs: spiderlike humanoid fantasy creatures, each with their own name and special characteristics. It is hard to believe there is no wire or any other support inside them. They are strong and in balance because they are made of woolen threads wound up so tight that they act as a kind of construction.

It was on this day that I met with Maurits Sterkenburg (1987) and his work for the first time and fortunately we have kept in touch ever since. The more I learned about him, the more I became fascinated by his creativity and his professional and serious artistic attitude. Maurits is a multitalented person. Not only is he working on his colourful Kolozaieken every day, he also makes animation films with music he composes himself and writes extraordinary poems. Recently, he started to paint as well.

**Fig. 1.**
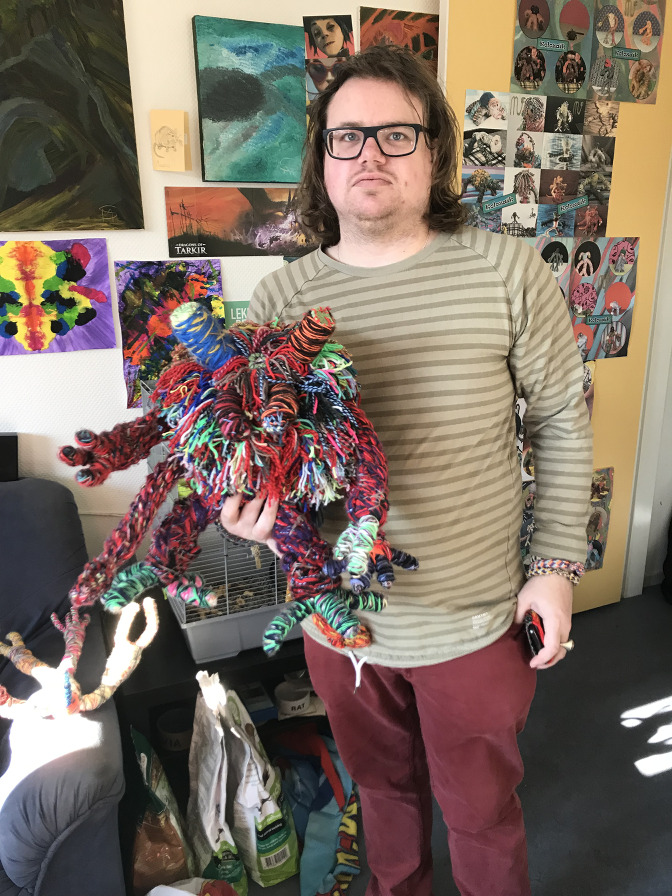
Maurits Sterkenburg.

Maurits expresses himself by means of several different media, and when you look closely at his oeuvre you will notice that his great drive is the need to artistically connect to the world around him. He is autistic and has suffered from psychosis in the past. He lives on his own in Veenendaal, a small city in the middle of the Netherlands. He has residential counselling and works not only at home but also in his studio in a squatter space where you can find more than 300 Kolozaieken. From an early age he was interested in animals, and the urge to create his monster-like woolen creatures originates from that time.

He knows them all by heart and gives them names. There is only one figure with five fingers and another seems to be crippled with legs that don't reach the ground. He talks about them with a love and tenderness as if they were his own children. He describes one of them as a rather annoying figure with multicoloured dreadlocks. He couldn't stay at Maurits's home any longer because he jumped off the cupboard in the hall every time Maurits walked by. ‘He is some kind of a mystery’ he explains.

**Fig. 2.**
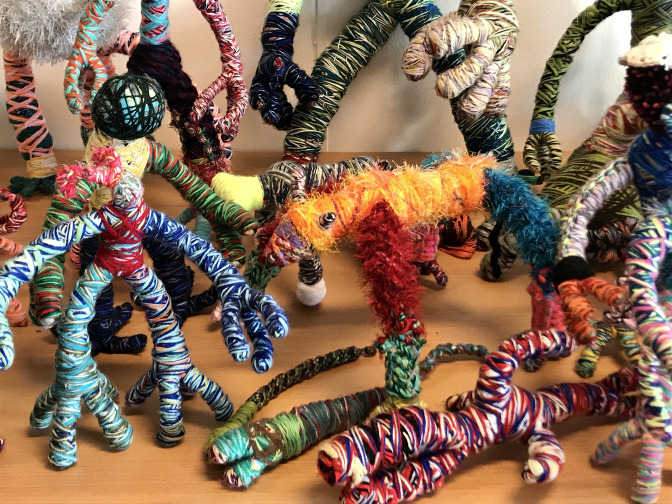
Kolozaieken.

**Fig. 3.**
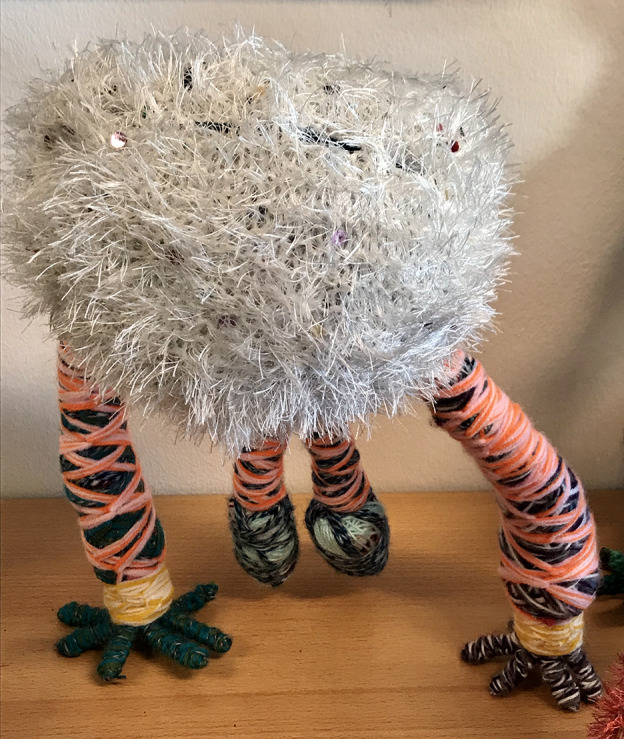
Kolozaieken.

**Fig. 4.**
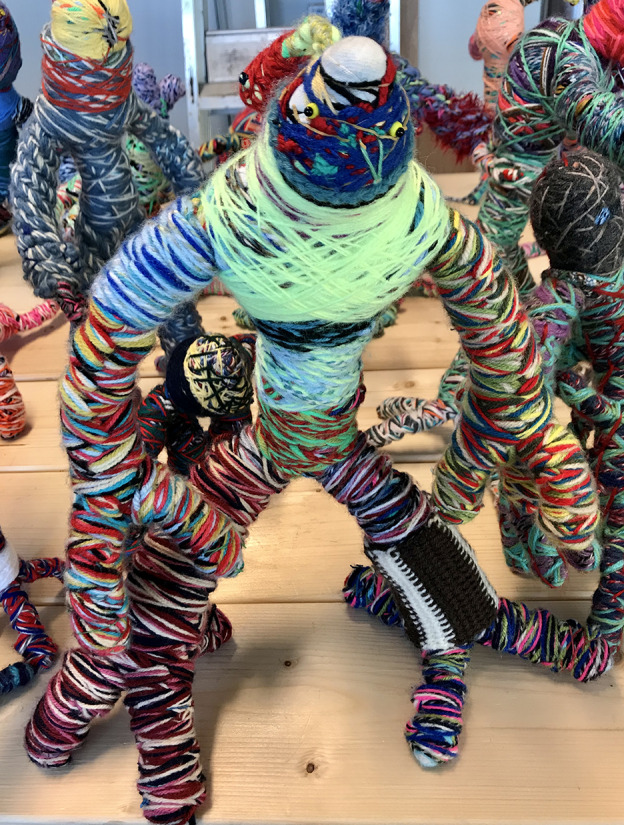
Kolozaieken.

The puppets are rather heavy because of the many layers of wool. ‘When I start, I don't know how they will turn out’ says Maurits. ‘They are more or less originating by themselves during the winding process’. He starts with three separate colourful threads that he braids or crochets together with his fingers. Because of the inner tension that he experiences, the threads are applied extremely tight.

Maurits is very aware of what he does. He is experienced and he makes his choices during the process very deliberately. Failures are cast aside in a separate ‘experimental’ bag. Still, his way of working remains a secret. He is often asked to lift a corner of the veil about his creative process or to do an instruction workshop but he remains secretive about his inventions and methods.

It is remarkable that his woolen friends still look like the outcome of a boyish mind. They develop slowly, when you look at their forms, they become more monumental nowadays and even more fantastic but somehow they still remind me of the prototypic monstrous robots from early science fiction movies. In his animation films his Kolozaieken are the main characters. They jump up and down to the music, often in front of a multicoloured textile background. His goal is to create a whole army of these ultra-positive woolen puppets and overwhelm the world with joy and happiness.

## His poetry

Maurits is a very intelligent person and that shows best in his poetry. Unfortunately, I cannot quote some of his recent poems here because they are in Dutch. The poems shown here in English are from a couple of years ago.

In his revealing poem ‘De ontdekking’ (‘The Discovery’) he talks about the fact that he will never fit in the standard idea of settling down and starting a family. He writes about the courage that he found to ascend from the depths of his mind and how he finally learned to deal with himself. He read this poem aloud during the opening ceremony of the Art Brut Biënnale (Hengelo, 2018) and a lot of people were truly moved by so much straightforward openness.

His phrases are authentic, original and meaningful. He is painting with words, colourful and intense. Working in the same way as he does while composing his music, he is constructing his texts very careful but determined. He produced a couple of small books all by himself with his poems, illustrated by images of the Kolozaieken. The poems follow the different stages in his development very consequently and are, in a way, witnesses of his critical view towards himself.

Striking is the humorous undertone in several of his poems. Serious matters like ‘being autistic’ are captured by Maurits in a dry reflective manner. He opposes raw daily reality to the consolation of daydreaming and subtle surreal fantasy. It feels like an invitation to peek into his mind for a short moment.

I am not a professor in literature but I know the history of art very well and many of Maurits's poems remind me of the DaDa movement and Surrealism. Like no other, he knows how to point out the absurd situations that he as a person with autism comes across every day. He grabs them in words, mirrors them, puts you on the wrong foot by using a little humour here and there and makes you feel a bit scratched and affected.

He plays with words and images and gives you a new view on reality. And that is in my opinion certainly one of the important aspects of art. I admire Maurits for his ongoing persistence. The way he inspires himself to get on with his creative plans: the making of the videos and books, the Kolozaieken, the music and the texts.

In his native city Veenendaal he is judged by who he is and seen as an outsider. He is not respected for his most interesting work and the artistic value he could add to the city's cultural life. Fortunately, the past 5 years brought him national and international recognition because people interested in Art Brut or Outsider Art immediately saw his qualities. He has shown his work in at Kunsthaus Kannen in Germany (September, 2018), and at Oeil Art in France (November, 2018), and meanwhile his work is bought by the Outsider Art Museum in Amsterdam: the highest honour for Art Brut artists in the Netherlands.

He is a good interpreter of his own poems and he is often asked to do a performance in combination with an exhibition. His voice must be heard, and his work certainly must be seen. His content is strong, he works with great passion and he has this big aura of optimism around him.

Once he wrote: ‘My imperfection is so perfect that you surely will freak out’. And I fully agree with that!

**The poems**

**they found me**

ink drips from my fingernail
into a puddle on the floor
I haven't been writing
for a long long time

the crusts forming on the walls
are filled with my wildest dreams
they sparkle and glow
in all sort of colours

I was just put in this room
others might call it a home
they like it when I decorate space
and there never was an agreement
from my side

**between blasts of water**

don't catch a cold
-just-
catch my drift
okay, well that was on me

just trying to get a good laugh
what clown do I need to beat up
before even thinking about
the guy that always gives me riddles

I just don't want this anymore
last a man who thinks he's a penguin
stabbed at me with an umbrella
then a dude in a weird suit
yelled hasta la vista, baby!
and ran off after he said
I'll be back

it's so confusing in here
the clown always asking me
why I am so serious
and I don't know the answer
because nobody likes me
even my clay dolls ignore my complaints

and I don't even know why I'm here
the guards are mean so I have to beat them
and then I always end up in here
in this white pillowed room

can someone get me out of here
before I go completely out of my mind

**there's no defense against it**

not gonna sugarcoat every message
but in some kind of way
I'm pretty fond on sugar

so I have a jar full of it
and I take it to the streets
while wearing a leather coat
as in trench coat

when talking to strangers
I give them a free sample
but only if they are sweet too
because…. just so my day is great
no what if's

and I hope that people will learn
from these little kind gestures
that they also can become
one of the many sweeties

**not just a desire**

being forced to this daily pill
I find my body in pain - so
I'm asking myself where my mind is at
where is it hanging out

what they both want
is not what they get
and that's just not a fair situation

I need trust

I need trust
from the people around me
so I can heal some stinking wounds
that have been there for far to long

## About the author

**Elvira van Eijl** is founder and curator of the International Art Brut Biënnale in Hengelo, the Netherlands. She is an experienced figure in developing contemporary art organisations, and lectures internationally as well as at the Moving Image and Fine Art Departments at the ArtEZ University of the Arts, Enschede, NL.

Carole Tansella, *Section Editor*
